# Minimally Invasive Tensiometry: A New Modality for Per-Operative Measurement of Medialization and Tension During Laparoscopic Hernia Surgery

**DOI:** 10.3389/jaws.2022.10850

**Published:** 2022-09-27

**Authors:** F. P. J. Den Hartog, E. F. Van Koten, J. J. Van Den Dobbelsteen, P. J. Tanis, M. Van Der Elst, A. L. A. Bloemendaal

**Affiliations:** ^1^ Department of Surgery, ErasmusMC, University Medical Center, Rotterdam, Netherlands; ^2^ Department of Biomechanical Engineering, Delft University of Technology, Delft, Netherlands; ^3^ Department of Surgery, Amsterdam UMC, University of Amsterdam, Amsterdam, Netherlands; ^4^ Department of Surgery, Reinier de Graaf Gasthuis, Delft, Netherlands

**Keywords:** hernia, minimally invasive, abdominal wall surgery, fascial tension, tensiometry

## Abstract

**Background:** Newly developed techniques for minimally invasive abdominal wall reconstruction (AWR) for complex ventral hernia are continuously evolving. In order to achieve hernia defect closure, the aponeurotic edges of the hernia defect need to be approximated. Currently, surgeons have no way to objectively measure and quantify the traction required to approximate these edges. This study presents minimally invasive tensiometry (MINT), a novel technology for measuring fascial tension, as well as initial experiences and results using it.

**Methods:** The MINT device was designed using rapid prototyping principles. It was designed as an add-on tool for any existing laparoscopic instrument, enabling objective assessment of abdominal wall tension by the use of a manually operated linear spring. Pre-clinical measurements of medialization at 10 and 20 N of tension during AWR were performed on fresh-frozen Post-Mortem Human Specimens (PMHS).

**Results:** Three specimens were included, and a total number of 36 measurements of medialization at three different levels of the abdominal wall were performed under structured and similar circumstances. Median total medialization with 20 Newton (N) of applied tension was 25 mm (mm) cranially, 37.5 mm at the umbilicus and 27.5 mm at the caudal level. The highest rate of medialization was seen at the umbilical level (2.25 mm/N).

**Conclusion:** MINT is a novel non-invasive technique, which allows surgeons to intraoperatively measure fascial tension when performing AWR. The MINT device is easy to use and reproduce. The next step is to start performing clinical measurements applying MINT during AWR.

## Introduction

There is an advent of minimally invasive techniques such as laparoscopic, robotic and hybrid techniques, for both complex and non-complex AWR. Outcomes are promising, with equal or lower recurrence rates compared to open surgical approaches, and lower rates of surgical site infection, seroma and hematoma (1,2,3,4,5). Additionally, due to less traumatic skin incisions, patients have less postoperative pain, a shorter length of stay and a more rapid return to physical activity such as sports and work (6,7,8). The ideal end situation during AWR is fascial closure, which is often augmented with mesh implantation, ideally in retro-rectus position (9). The abdominal wall defect (hernia) may be too large to close without the aid of techniques to gain additional medialization, such as retrorectus dissection combined with anterior or posterior component separation techniques (CST). The main difficulty is the tension in the lateral abdominal wall, which needs to be overwon to achieve permanent closure of the defect. The force, or traction, needed to overcome the myofascial tension can vary widely between hernias, mainly depending on patient characteristics, recurrence status and previous mesh placement. High tension on the approximated aponeurotic edges is associated with ischaemia and can lead to incisional hernia (IH) recurrence (10). Intra-abdominal hypertension and abdominal compartment syndrome, which can have devastating consequences, have also been described after larger hernia repairs (11). Additionally, in a prior experimental study, no correlation was found between hernia width and abdominal wall tension, meaning that the size of the defect alone is not an indicator for the expected tension on the abdominal wall (12).

Several methods have been found in literature to objectively assess abdominal wall tension during open repair, but surgeons have no way to objectively measure either of these two factors, medialization and tension, during minimally invasive surgery (12, 13). Minimally invasive tensiometry is being developed in order to fulfil this role. MINT is an add-on tool for existing laparoscopic instruments, which enables an objective assessment of abdominal wall tension by the use of a linear spring. Due to the many difficulties that come with the lengthy and costly development and regulatory approval of novel laparoscopic instruments, the choice was made to focus on the design of a simple, easy to administer add-on instrument that can be used with several different manufacturer’s laparoscopic devices all over the world. This decreases time to value and speeds up the process of collecting relevant data for further research.

This study presents a brief description of this new measurement device, as well as initial experiences and results using MINT on fresh-frozen PMHS.

## Methods

### MINT Device Development

Primarily, the device needed to enable quick and easy measurement of tension without significantly changing or elongating the procedure. To enable universal applicability, the device design needed to comply with several different shapes and sizes of laparoscopic instruments, without interfering with their functionality and safety. Additionally, the device needed to reliably, accurately and safely measure the tension exerted on the tissue during repair of the abdominal wall defect. Lastly, the 3D printed device has to be easy to fabricate on a large scale and should be sterilized without altering any functional status.

Rigid clamping of the laparoscopic instrument needed to be enabled whilst resisting the pulling force (traction) necessary to close the defect. Thus, it was chosen to use a clamping mechanism relying on mechanical fastening. Due to safety and functionality restraints of laparoscopic instruments, the chosen point of attachment was the handle. Keeping requirements regarding safety, simplicity and sterility in mind, it was decided to go for a purely mechanical force sensor using a linear spring scale, inspired by methods used in open repair (12, 13). Force is measured by assessing the elongation of the spring as a result of the pulling force (Hooke’s law). The most important part of this design aspect is the stiffness of the spring. On one hand it needs to be stiff enough to give an accurate representation of the force exerted on it, on the other hand its elongation needs to be large enough so the surgeon can quickly and clearly assess the elongation on a scale during the procedure. The spring requires a working range between 10 N and 60 N, the upper limit was based on a literature review (14). The handlebar used to operate the device depicts the force exerted on the instrument during use.

Rapid prototyping was used to manufacture the device as it brings many benefits related to manufacturing time, design possibilities, assembly, and costs. The material of choice was carbon fiber reinforced polyamide-12 (PA-12, “Onyx™”, created by Markforged), as PA-12 is not only known for its tensile strength, impact strength and toughness, it is also easier to sterilize than the frequently used polylactic acid (PLA). PA-12 is recommended for making parts of surgical instruments that need to be sterilizable by autoclaving (15). Results indicated that the prototype withstands at least five cycles of steam sterilization (14).

**FIGURE 1:**
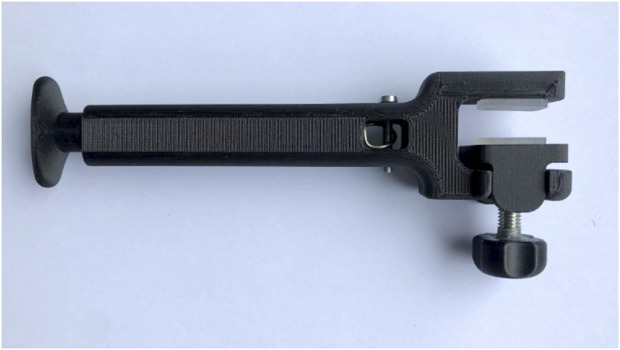
The MINT device’s C-clamp design.

**FIGURE 2:**
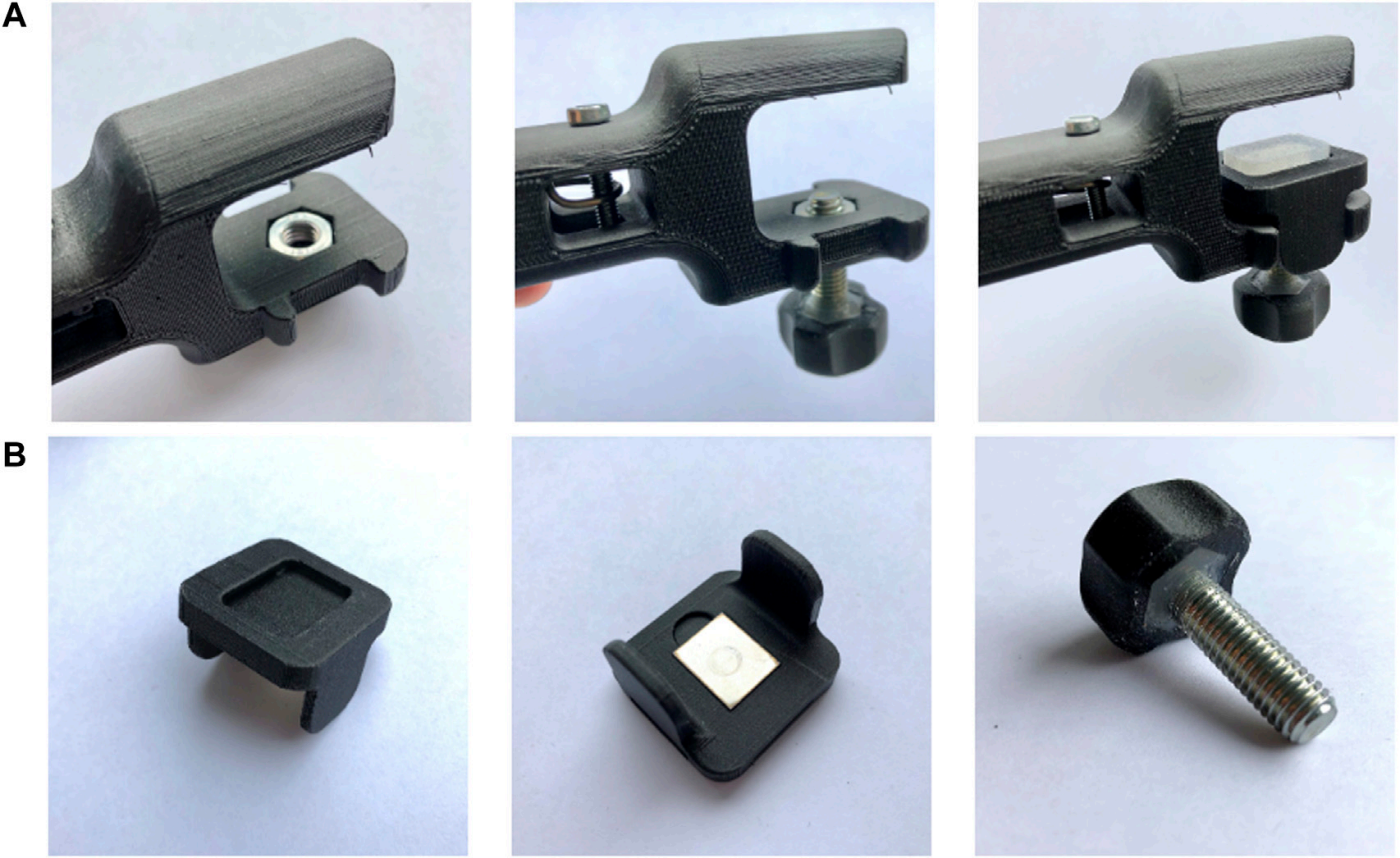
The clamping/adjustment bolt-and-nut mechanism (**(A)**: Assembly of device, **(B)**: Detailed view of the block that creates the contact surface between the clamped instrument and the device).

The clamp design was inspired by the well-known C-clamp (Figure 1). It consists of a frame, shaped like a C, which forms into a slender, hollow rod-shaped segment that houses the spring and handle of the device. A block is used to enable clamping of devices with variable thickness (Figure 2). This block was designed so that it could only move up and down without moving forward, backward or sideways. The bulges on the lower beam of the frame created slots for the walls of the block to slide through whilst the device was being used (Figure 2). The two contact surfaces contain cutouts that house silicon pads. These did not only account for different handle shapes by cushioning the handle, but they also protected the handle from breaking during clamping and increased friction between the handle and the clamp (Figure 2). At the bottom of the block a cutout was created in which a stainless steel plate was placed (Figure 2). The sheet made sure that the bolt did not damage the block while adjusting the device and fastening the clamp. The slender rod shaped part of the housing contains a hole throughout its entire length.

**FIGURE 3:**
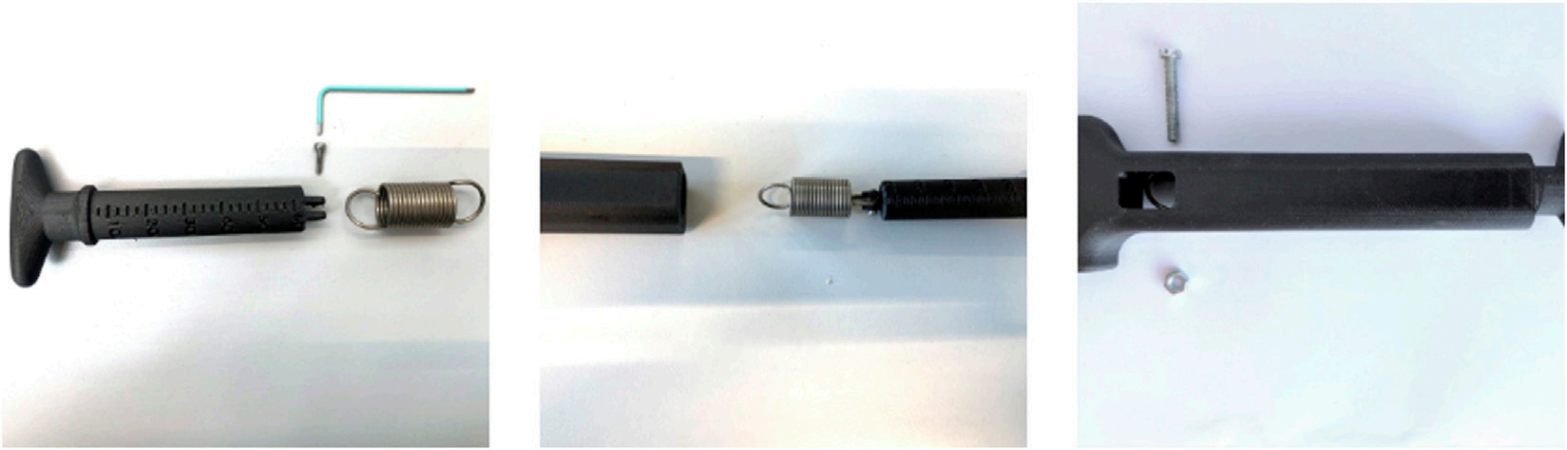
The spring/handle assembly (left: disassembled, analog scale of 10–60 N visible, middle: partially assembled, right: assembled, before locking spring with bolt and nut).

The handle, connected to the spring, is inserted here and connected to the housing using a pin (Figure 3). Holes on the side of the housing enabled easy assembly of the device. The device was designed in such a way that the pre-tension of the spring is taken into account whilst using the device. During operation, the handle of the device will be pulled out of the housing, displaying the measured force necessary to do so on an analog scale (10–60 N). The scaling was created after the spring constant was validated using weights. It was assumed that most force measurements would be within the working range of 10–60 N, as lower forces point to direct closure and higher forces will most likely indicate component separation.

### Specimen Selection

Experiments were performed on fresh-frozen PMHS in the anatomical lab of the Erasmus University Medical Center, department of Anatomy and Neuroscience. Specimens were obtained from the university’s body donations program, wherein donors consented to donation for scientific purposes. As these experiments were performed on PMHS and not on live human or animal subjects, per local regulations, no formal ethical committee approval was required. Due to Dutch privacy law, specimens’ medical histories were unavailable, but specimens with visible abdominal scarring, which might allude to previous abdominal pathology, were excluded.

### Experimental Procedure

**FIGURE 4:**
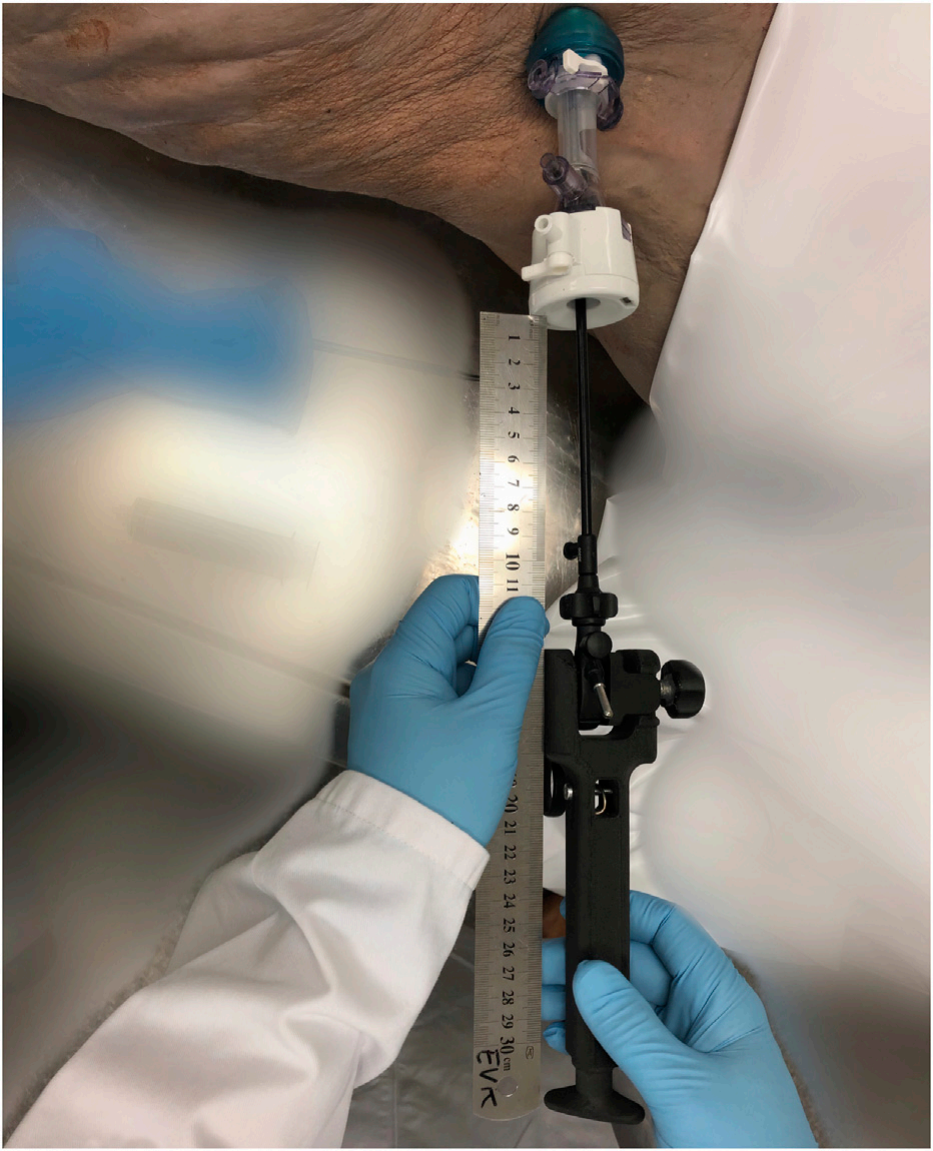
Fully assembled MINT device with laparoscopic instrument clamped in.

**FIGURE 5:**
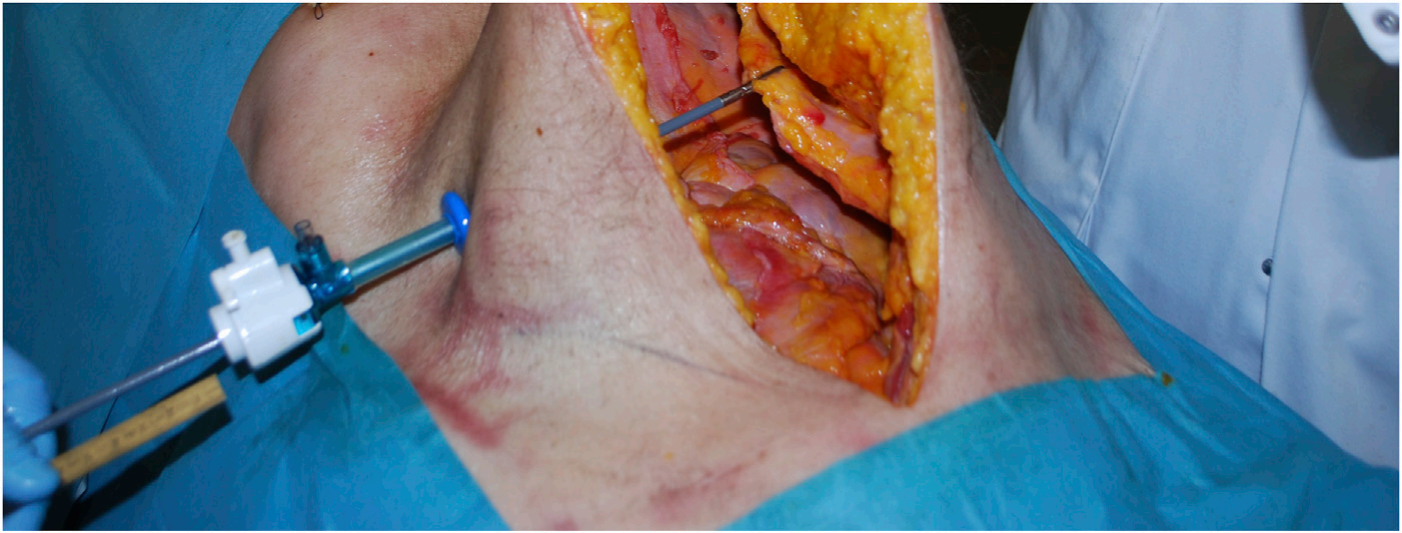
Tissue being grasped with laparoscopic instrument.

Experiments were performed during a surgical skills training session, where experienced hernia surgeons educated other surgeons on the posterior CST-TAR. After completion of the posterior CST, a single 5-mm trocar was inserted at the umbilical level, as laterally as possible. Through this trocar, atraumatic fenestrated grasping forceps (Symmetry Surgical™ Access 32-5117R) were inserted and the contralateral anterior rectus sheath was grasped (Figures 4, 5). Consequently, the MINT device was attached to the handle of the instrument, by turning the rotation knob until sturdy clamping is achieved. The surgeon applied tension to the grip in order to pull the edge of the defect to the mid-line of the abdomen. Simultaneously, the displacement of the device relative to the trocar was read out using a ruler.

Measurements of the abdominal wall were taken at three levels: cranial, umbilical and caudal, meaning that the anterior rectus sheath was grasped at these three levels. The tissue was pulled taut first, without visually straining the tissue. Then, tension was applied, and medialization was measured at 10 N and 20 N. Afterwards, the trocar was moved to the contralateral side and measurements were repeated for the other side of the abdominal wall. This allowed for a total of 12 measurements per specimen. After having acquired enough results, the MINT device was detached.

### Statistical Analysis

Data was collected in a spreadsheet. Medializations in millimetres and tensions in Newton were summarized and a median was calculated. For each of the three measurement levels (cranial, umbilical and caudal), between 0 to 10 and 10–20 N of tension, the medialization gained for every Newton of applied tension was calculated (mm/N), as well as the inverse (N/mm). No statistical tests were performed, presented results are of descriptive nature.

## Results

Three fresh-frozen post-mortem human specimens were included. All were males, over the age of 70. In total, 36 separate measurements were performed: 12 in each specimen.

### Medialization

**FIGURE 6:**
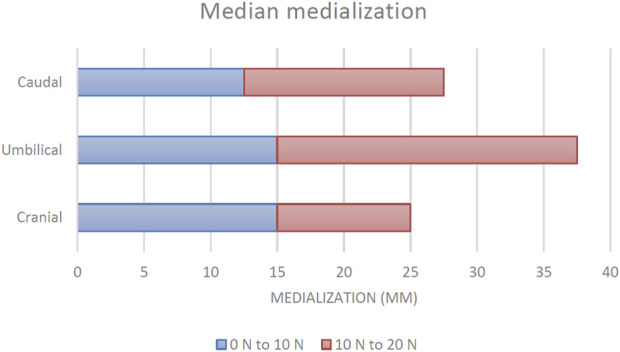
Median medialization at three levels of the abdominal wall (n = 12 measurements per level). Source data can be found in Supplementary Table S1.

The median amount of medialization across the three included specimens was determined at cranial, umbilical and caudal levels. Figure 6 displays these results.

In the first 10 N of applied tension, a median increase in medialization of 15 mm was found cranially. Umbilically, this was also 15 mm. At the caudal level, 12.5 mm was observed. When a total of 20 N was applied, an additional median medialization of 10 mm was observed at the cranial level. At the umbilicus, this was 22.5 mm. Caudally, the additional median medialization was 15 mm.

Total medialization with 20 N of applied tension was 25 mm cranially, 37.5 mm at the umbilicus and 27.5 mm at the caudal level. Additionally, the relationship between tension and medialization was calculated and it is displayed in Table 1. The lowest amount of medialization per N was seen at the cranial level, followed by the caudal level and the highest amount was seen umbilically.

**TABLE 1:** Median rate medialization at different levels of the abdominal wall.

	Cranial	Umbilical	Caudal
0 N–10 N	1.5 mm/N	1.5 mm/N	1.25 mm/N
	0.67 N/mm	0.67 N/mm	0.80 N/mm
10 N–20 N	1 mm/N	2.25 mm/N	1.5 mm/N
	1 N/mm	0.44 N/mm	0.67 N/mm

## Discussion

MINT was developed in order to enable hernia surgeons performing minimally invasive surgery to intra-operatively measure whether they can achieve adequate medialization of the rectus sheath, and to record what amount of tension is required to do so. In these first preclinical experiments using MINT after posterior CST, median medialization with 10 N of tension was between 12.5 and 15 mm. At 20 N of tension, a medialization between 25 and 37.5 mm was measured. Ultimately, MINT could aid in decision making on whether additional CST or intra-operative fascial traction will be necessary, or whether retro-rectus dissection will suffice.

One of the key aspects of MINT is the non-invasive way of measuring. No additional devices are introduced into the patient’s abdomen. Surgeons can grasp the rectus sheath tissue using the instruments that they are already familiar with and that they would typically use. The MINT device is merely clamped to the handle of the laparoscopic instrument of choice in order to achieve measurements. Measurements can be made rapidly. While the time to measure was not included as a parameter in the present study, anecdotally, measurements cost approximately 30 seconds to 2 minutes, which was mainly dependent on finding the right position to grasp the tissue.

In anatomical experiments, performing retro-rectus dissection and posterior CST has shown to yield 5.8 – 9.9 cm of absolute anterior rectus sheath medialization (16, 17). The medialization measured during the present experiments is much lower at approximately 2 cm, however it cannot be compared because it does not represent absolute medialization. There is a lack of a reference point in these measurements, because before any resistance is experienced from pulling the tissue, so before it is pulled taut, there is already significant medialization achieved, but this medialization was not measured.

A key difference between fresh frozen and *in-vivo* experiments is muscle tension, of course completely absent in a cadaver. Findings in our study can therefore not simply be extrapolated to patients. However, during AWR, muscle relaxants are administered perioperatively in order to facilitate closure. In that respect, a fresh-frozen model represents the ‘ideal’ situation, which is one without any muscle tension. In living humans, muscle tension will be higher than the tensions found in the present experiments, due to the remaining muscle activity.

Not every aspect of the MINT device was tested in this study. For instance, device handling has not been tested yet, however this is an aspect of MINT that is under active development. Eventually, this will be structurally evaluated by surgeons. Further improvements to the MINT device are ongoing. An example of improvements that are being worked on is to reduce the number of components that could potentially loosen or even drop, whilst retaining the ability to sterilize the device. Clamping of the device to the variety of laparoscopic instruments that exist needs to be completely reliable and secure.

**FIGURE 7:**
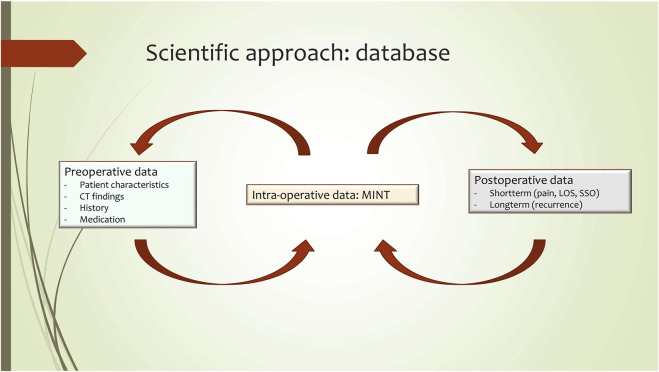
MINT scientific approach.

The first goal of AWR is primary closure of the anterior fascia. Presently, there is no way to preoperatively determine whether, for example, posterior CST or intraoperative fascial traction will be necessary in order to achieve adequate fascial medialization and closure. MINT could be used to intraoperatively measure fascial tension and this data should then be saved, along with patient characteristics, medical history, medication use and diagnostic results (e.g. CT-scans), in a database (Figure 7). Such a database can subsequently be used to develop a prediction model, which might be of additive value in deciding on the most appropriate surgical technique in the preoperative setting. Outcomes such as postoperative complications (surgical site infection, seroma, hematoma), pain, length of stay and recurrence could be evaluated in future comparative studies with and without using the additional preoperative information and the intraoperative use of the MINT device. Because MINT tensiometry is non-invasive and takes little effort, getting surgeons to participate in clinical evaluation studies is expected to be relatively easy. Already, several Dutch hernia surgeons have expressed interest in participating.

## Conclusion

MINT is a novel non-invasive measurement technique, which allows surgeons to intraoperatively measure fascial tension when performing abdominal wall reconstruction. The MINT device is undergoing active development. The next clinical step is to start applying MINT in studies across centres in the Netherlands, creating the foundation for a database that could allow preoperative predictions with regard to which surgical technique to use and what outcomes to expect for a specific patient. Ultimately, this leads to patient-tailored operation planning and a reduced hernia recurrence rate.

## Data Availability

The original contributions presented in the study are included in the article/Supplementary Material, further inquiries can be directed to the corresponding author.
